# A Universal and Single-Step (De)Molding Sorting Chip Integrating Inertial and Deterministic Lateral Displacement Units

**DOI:** 10.3390/bioengineering12121326

**Published:** 2025-12-05

**Authors:** Yifan Guo, Xiaoyu Qu, Zhaogang Dong, Mengmeng Xiao, Jingjing Xu

**Affiliations:** 1 Shandong Key Laboratory of Next-Generation Semiconductor Technology and Systems, School of Integrated Circuits, Shandong University, Jinan 250100, China; 2 Department of Clinical Laboratory, Qilu Hospital of Shandong University, Jinan 250100, China; 3 Key Laboratory for the Physics and Chemistry of Nanodevices and Center for Carbon-Based Electronics, Department of Electronics, Peking University, Beijing 100871, China

**Keywords:** microfluidic chip, cell sorting, inertial sorting, deterministic lateral displacement, high sorting rate

## Abstract

Serum tests are valuable sources of information for disease diagnosis. Conventional whole blood cell separation requires many processing steps, including centrifugation, fractionation, lysis, and dilution, and is therefore complex and time consuming. To address the need for the efficient separation of blood cells for on-chip rapid serum assays, we developed a microfluidic chip integrating inertial sorting and deterministic lateral displacement. This chip consists of a helical structure and a deterministic lateral displacement triangular microcolumn array for rapid and efficient separation of blood cells from whole blood samples. After separation, the supernatant is extracted at the exit for subsequent testing or directed to serum test units directly integrated in the chip. Here, the laminar flow and transport modules are coupled using finite element analysis for both multi-component and discrete-phase physical fields to simulate blood flow characteristics in the chip. The influences of flow rate and flux ratio on the sorting efficiency of blood cells were also discussed. Simulation results determined that the microfluidic chip designed in this research can achieve a cell sorting efficiency greater than 98% at suitable flow rates. Experimental results similarly achieved a high sorting effect of above 96%. Therefore, this blood cell sorting microfluidic chip shows strong potential for rapid serum testing applications and can be integrated as a stand-alone blood cell sorting module for various on-chip serum testing systems used to diagnose diseases.

## 1. Introduction

Many human biochemical indicators can be measured using serum tests. Blood cell interference is an important factor affecting the accuracy and precision of serum testing. The traditional multi-step serum testing protocols are generally unable to meet urgent demands for the rapid diagnosis of infectious diseases such as during the COVID-19 pandemic [1,2]. In contrast, on-chip diagnostic techniques are currently the fastest methods of detecting infectious diseases, and microfluidic sorting technology is the most important for achieving serum and blood cell separation in on-chip systems [3,4]. According to the working principle, microfluidic sorting technologies can be divided into active sorting techniques [5,6], which have the disadvantages of easy clogging, complex operation and fabrication, low sorting efficiency and cell activity, etc., and passive sorting techniques that utilize the differences in fluid forces acting on cells of different sizes in the fluid. Common structures used to achieve this in microfluidic chips include inertial spirals [7], entrained-flow separation [8], microfiltration sorting [9], or deterministic lateral displacement [10]. These structures are considered to be a promising technology for cell separation and extraction due to their high throughput, label-free and field-free operation, and due to their simple channel structure [11].

However, it is difficult for a single sorting technique to simultaneously show high cell throughput, high sorting efficiency, and high cell viability, three key sorting requirements. To address the widely recognized problem of sorting efficiency, researchers have developed multi-stage sorting microfluidic chips. These can include multiple iterations of a single technique or multiple techniques integrated together [12,13,14]. The efficiency of single-stage sorting typically falls within the 44–97% range, and the sorting efficiency of multi-stage designs has been reported to generally improve to a range of 85–99%. Abdulla et al. designed a two-stage spiral sorting chip in which both the primary and secondary inlets are located on the outer side of the spiral. This reduces the number of catheters required, and a single-entry spiral with a small cross-sectional size can be used to separate blood cells from tumor cells in the primary stage, and a double-entry spiral with a larger cross-sectional size is then used to separate small A549 lung cancer cells from larger MCF-7 breast cancer cells in the secondary stage. Thus, this two-stage chip can achieve continuous separation of erythrocytes, leukocytes, A549, and MCF-7 cells. Moreover, it achieved separation efficiencies for A549 and MCF-7 of 80.75% and 73.75%, respectively [15]. Another multi-stage design by Ozkumur et al. integrated DLD, inertial spiral, and magnetic sorting techniques [16]. The primary DLD structure was used to remove all other blood components except for larger bead-labeled cells; the secondary asymmetric sinusoidal flow channel was used to focus larger cells into a single contour. A tertiary magnetic sorting structure was used to filter out bead-labeled leukocytes to obtain high-purity tumor cell samples. Using this chip, the authors were able to achieve efficient separation with a recovery rate of human breast cancer cells of 98.6 ± 4.3% and a capture rate of human prostate PC3-9 cancer cells of 89.7 ± 4.5%. This structure is highly costly in terms of chip preparation and reagent consumption. Moreover, multi-stage integration can increase chip flow resistance, and challenges related to chip packaging and cell viability remain. Thus, the design of the final chip structure should weigh each of these factors carefully [17,18].

Due to increasing demand for high sorting efficiency and rapid assays, it is difficult for existing blood cell sorting devices to simultaneously achieve high sorting efficiency, sufficient throughput, and low cost. In this paper, we integrate inertial spiral and DLD sorting technologies on a single chip, in order to improve overall sorting performance. This study uses simulations to continuously improve blood cell sorting chip design and to analyze the effects of channel structure, medium mixing ratio, and fluid flow rate, among other factors on cell sorting. Using these data, we determine the best sorting parameters and provide theoretical verification for many parameters that are difficult to evaluate experimentally. Therefore, the aim of this paper is to ensure high sorting efficiency by examining model design and simulation parameters and determining how they affect the cell sorting process.

## 2. Methods

In this study, we used Ansys Fluent^®^ software to construct a two-dimensional geometric model of a microfluidic chip with integrated inertial and DLD sorting. The adequate mixing of blood samples and buffers in this simulation is to ensure a uniform distribution of multi-component fluids prior to cell sorting. The difference between the mixed fluid density and viscosity coefficient of whole blood samples is small. With an average cell density of ρ = 1.05 × 10^3^ kg/m^3^ and a buffer density of ρ = 1.0 × 10^3^ kg/m^3^, we set the average cell kinetic viscosity as μ = 0.3 × 10^−2^ Pa·s and the buffer viscosity as μ = 0.1 × 10^−2^ Pa·s for the simulation; thus, the Reynolds number of the buffer mixture of blood in this study was approximately 0.003–3 (i.e., Re = ρUf Dh/μ, where ρ is the fluid density, Uf is the average flow rate, μ is the dynamic viscosity, and the hydraulic diameter Dh = 2wh/(w + h)). In the process of simulated cell sorting, we introduced a total of 12,100 particles; the ratio of white blood cells, red blood cells, and platelets was 1:1000:40, the same ratio in blood. Five cells were released at each time step. In addition, the characteristic scale of fluid flow is between the micron and millimeter orders; thus, the mean-free-path approximation was selected to solve hydrodynamic control equations. This, in turn, helps simulate blood flow in a microfluidic chip. As the subject of investigation in this system, blood is a non-Newtonian fluid and is typically modeled as an incompressible flow without heat transfer between fluid components. Therefore, this research does not need to consider the law of conservation of energy, but only the laws of conservation of momentum and conservation of mass.

### 2.1. Geometric Model

**Figure 1:**
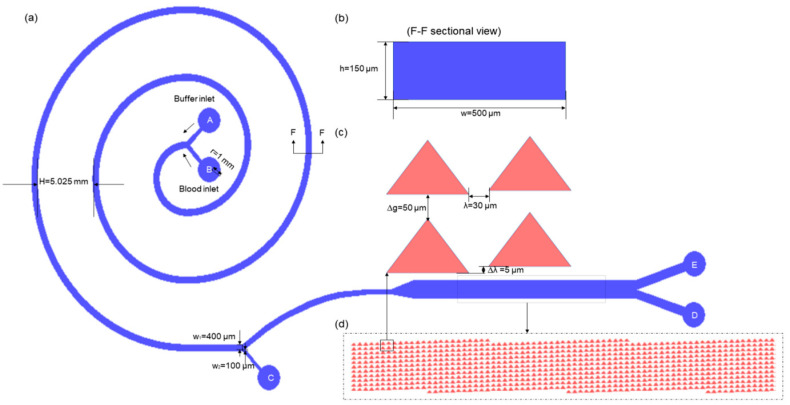
Geometric model of the microfluidic chip for blood cell sorting. (**a**) Overall design of the flow channel; (**b**) schematic cross-section of the inertial spiral flow channel at F-F position; (**c**) schematic diagram of the triangular microcolumn array dislocation mode in the DLD module; (**d**) arrangement diagram of the triangular microcolumns in the DLD module.

The blood cell sorting chip consists of two inlets, a main flow channel, and three outlets, as shown in Figure 1a. The two inlets are the whole blood sample inlet and the buffer inlet. The main flow channel consists of two connected sorting modules to improve blood cell sorting efficiency through a double-sorting-channel design. The three outlets include the spiral outlet C located at the end of the spiral channel, the secondary outlet D, and the serum outlet E located at the end of the DLD channel.

#### 2.1.1. Spiral Channel Parameter Design

We used inertial-flow-related physical properties to inertia to model a chip capable of efficient cell sorting. The specific design parameters of the spiral flow channel model are shown in Figure 1a,b. The radius of both inlets and outlets are 1.0 mm, the width of the rectangular cross-section of the spiral flow channel is 500 μm, and its height is 150 μm; the initial radius (R_0_) is 2.5 mm, the spacing between the flow channel (H) is 5.025 mm, the total length of the three turns is 22.5 mm, and the channel widths of the upper and lower outlets of the spiral flow channels W_1_ and W_2_ are 0.4 mm and 0.1 mm, respectively. We set the overall simulation environment to an incompressible flow model to better simulate the effects of flow rate and injection concentration ratio on sorting performance.

Buffer and blood samples were injected from inlets A and B, respectively, and the dimensionless concentrations of the two injection streams at the inlet were 0 for the liquid at the A inlet and 1 for the liquid at the B inlet.

#### 2.1.2. DLD Channel Parameter Design

To address the insufficient sorting accuracy of the spiral channel—particularly its difficulty in separating smaller platelets—a DLD structure, which provides higher separation resolution for cells of different sizes, was introduced to complement the spiral-channel module. We introduced the sample flow at the exit of the upper spiral channel into the lower DLD module. Given that triangular microcolumns have displayed superior performance and lower flow resistance, triangular micropillars were incorporated into the DLD module to enhance cell sorting efficiency.

The structural design of the DLD module is shown in Figure 1c,d. After a preliminary investigation of DLD displacement parameters and pre-simulation adjustments, we determined that the vertical distance (Δg) between adjacent micro-pillars should be 50 μm, the horizontal distance (Δλ) between adjacent micro-pillars should be 30 μm, and the dislocation offset parameter (λ) was set to 5 μm [6,19]. The main channel of the DLD module consists of a periodic array of triangular micro-pillars, with each array containing 24 × 11 triangular micro-pillars. The spacing of the microcolumns was determined based on the size of the target particles to be sorted. Among the cell sorting targets in healthy human blood, platelets are typically 2–4 μm in diameter, erythrocytes 5–7 μm, and leukocytes 10–18 μm. To sort platelets as efficiently as possible while still ensuring a high cell sorting efficiency, the critical size of DLD (Dc) was determined to be 2 μm. Cells larger than the critical size are displaced laterally, while cells smaller than the critical size will follow their original trajectory. For ε = 1/11, cells larger than the critical size are theoretically deflected to the lowermost side of the flow channel only after eleven cycles of movement in the microcolumn array. Thus, the length of the microcolumn array ensures that high sorting efficiency can be achieved despite the effects of collision, extrusion, and variability among cells, preventing larger cells from moving between microcolumns.

### 2.2. Simulation Settings

Using ICEM CFD 2021 R1^®^ software, a 2D model with integrated inertia and DLD was generated, the boundary conditions were set, and the model area was divided into sub-regions for subsequent meshing. The simulation process focuses not only on the vortices or trajectory changes of the cells in the main flow channel and each outlet but also on changes in their rotational motion and the potential for tail flow after the collision between the cells and the micropillars. Moreover, this simulation will calculate the grid for areas where the cell motion state will change and avoids allocating computational resources to regions with low precision requirements; this prioritization method will improve simulation speed.

After setting the minimum cell size to 0.005 mm and the maximum cell size to 0.699 mm and refining the mesh, the 2D model contained a total of 44,949 triangular mesh cells with a mesh quality of 0.22 or more. Next, a model in which the mesh quality met all simulation conditions was imported into the fluidics computing software Ansys Fluent 2021 R1^®^ for simulation setup and calculation. The two inlets were set as the flow-rate inlets, the outlet boundary condition was set as the pressure outlet, and the outlet pressure was set to 0 Pa (in order that the open outlet would be approximately one standard atmosphere, the simulation must set the static pressure here to 0 Pa). The wall condition of the main flow channel was set to a rebound condition.

To obtain accurate motion and sorting performance data for the model, discrete-phase and multi-component models were introduced for investigation of the effects of different inlet flow rates on total sorting efficiency, and the most appropriate solver was chosen to obtain accurate simulation results. A discretized coupling approach was used, applying the easily convergent, first-order accurate upwind scheme to solve the Navier–Stokes equations for the particle-free flow field. In addition, the SIMPLEC(SIMPLE-Consistent) algorithm, which enables rapid convergence for straightforward problems, was used to solve the pressure and velocity coupling problem and perform iterative calculations. After the iterative results converged, the complete dataset of the discrete-phase model was extracted. Finally, steady-state simulation analysis was conducted, and the results were processed to characterize the flow field.

### 2.3. Fabrication and Experiments

The chips were fabricated with the micro-nano processing techniques. The specific process included preparation of the mold for microfluidic channel and bonding. The former stage involved the cleaning of substrate, spin-coating and pre-baking of photoresist (SU-8 2075), UV photolithography, post-baking, and development. The prepolymer was poured on the channel mold, followed by a curing process to prepare the PDMS block with designed microfluidic channels. The PDMS block was bonded to a glass substrate to complete the chip fabrication. Polystyrene spheres and PBS buffer solution were injected into the chip. By adjusting the injection speed of the syringe pump and replacing different injection flux ratios, the sorting efficiency of non-identical-sized particles under different conditions was further verified.

### 2.4. Force Analysis in Spiral Channel

Plasma is a Newtonian fluid, but blood containing a large number of red blood cells is a viscous non-Newtonian fluid that satisfies the Newtonian constitutive equation only when diluted. Therefore, in the simulation, we selected a pressure-based solver with momentum and pressure as the main variables, which is often used for incompressible fluids such as blood. After the solver was configured, the solution of the fluid domain is to calculate the Navier–Stokes equation. Since the density difference between the cells and the surrounding blood is not large, the effect of particles on the fluid is neglected to simplify the computation. We supplemented the relevant methods and specific information in the article and highlighted it [20].

(1) $$m_{p}\frac{dV_{p}}{d_{t}}=F_{G}+F_{BA}+F_{D}+F_{B}+F_{VM}+F_{L}$$

Discrete particles move in a continuous fluid medium, and the forces affecting the acceleration of the particles are caused by the velocity difference between the particles and the displacement of the particles with respect to the fluid; the specific calculation formula is as follows:

(2) $$m_{p}\frac{dV_{p}}{d_{t}}=F_{G}+F_{BA}+\frac{18\mu }{\rho_{p}a^{2}}\frac{pm_{p}\left(V-V_{p}\right)}{24}+\frac{gm_{p}\left(\rho_{p}-\rho \right)}{\rho_{p}}+\frac{1}{2}\frac{\rho }{\rho_{p}}\frac{m_{p}d\left(V-V_{p}\right)}{d_{t}}+F_{L},\;\;$$

where t is the time, V_p_ is the velocity vector of the particle, V is the velocity of the particle, g is the acceleration of gravity, and ρ_p_ is the particle density. Equation (2) is derived from Newton’s second law of motion by balancing the integral forces. On the right side of the equation are the negligible gravity and Bassett forces, the drag caused by the interaction between the continuous and discrete phases on the particles, the virtual mass force that accelerates the fluid around the particles, and the acceleration caused by the inertial lift F_L_.

The trajectory of particles in the spiral channel is mainly affected by inertia lift force (F_L_) and Dean drag force (F_D_). The relationship between these two forces affects the net force acting on the particles and even their equilibrium position within the flow channel. The proportional relationship between the two forces is as follows:

(3) $$\frac{F_{L}}{F_{D}}\sim \frac{1}{\delta }\left(\frac{a}{D_{h}}\right)R_{e}$$

When the ratio approaches zero, Dean drag forces play a dominant role, and particles flow with Dean flow in the spiral channel, leading to the phenomenon of non-aggregation of particles. When the ratio tends to infinity, the inertial lift force becomes dominant, and the motion state and agglomeration of particles in the spiral channel are similar to those in the straight channel. When the ratio is close to one, the two forces act together on the particles in the spiral channel, causing them to focus at an equilibrium position. Therefore, based on this theory, we used the user-defined function in Fluent to add the effect of inertial lift [21,22].

### 2.5. Data Calculation

The number of cells collected at the lower outlet of the spiral channel and the number of cells collected from all inlets were counted to calculate the first stage sorting efficiency of the spiral channel. Here, we defined the total sorting efficiency as η and set η_1_ as the first level sorting efficiency (determined by the inertial spiral channel) and η_2_ as the second level sorting efficiency (determined by the DLD channel). We also defined the number of cells collected from all inlets as Q and set Q_1_ as the number of cells collected from the lower outlet (C) of the spiral channel and Q_2_ as the number of cells collected from the lower outlet (D) of the DLD.

The first level of sorting efficiency η_1_, which also represents the sorting efficiency of the spiral module alone, is the ratio of the number of cells collected from outlet C, Q_1_, to the number of cells entering the inlets defined as Q:

(4) $$\eta_{1}=\left(\frac{Q_{1}}{Q}\right)\times 100\%$$

The second level of sorting efficiency η_2_ is defined with the ratio of the number of cells collected from outlet D, Q_2_, to the number of cells entering the inlets, Q:

(5) $$\eta_{2}=\left(\frac{Q_{2}}{Q}\right)\times 100\%$$

The sorting efficiency of DLD, named as η_3_, is the ratio of the number of cells collected at outlet D, Q_2_, to the number of cells collected from the DLD inlet, Q-Q_1_:

(6) $$\eta_{3}=\left(\frac{Q_{2}}{Q-Q_{1}}\right)\times 100\%$$

As a result, the total sorting efficiency can be calculated as follows:

(7) $$\eta =\eta_{1}+\eta_{2}$$

## 3. Results and Discussion

### 3.1. Basic Structure and Performance of the Chip

The blood cell sorting chip designed in this study integrates inertial spiral and DLD sorting technologies in a two-stage design. This facilitates the continuous flow of fluids in both main streams to achieve two types of sorting simultaneously, thereby improving sorting efficiency and reducing loss during sample transfer.

**Figure 2:**
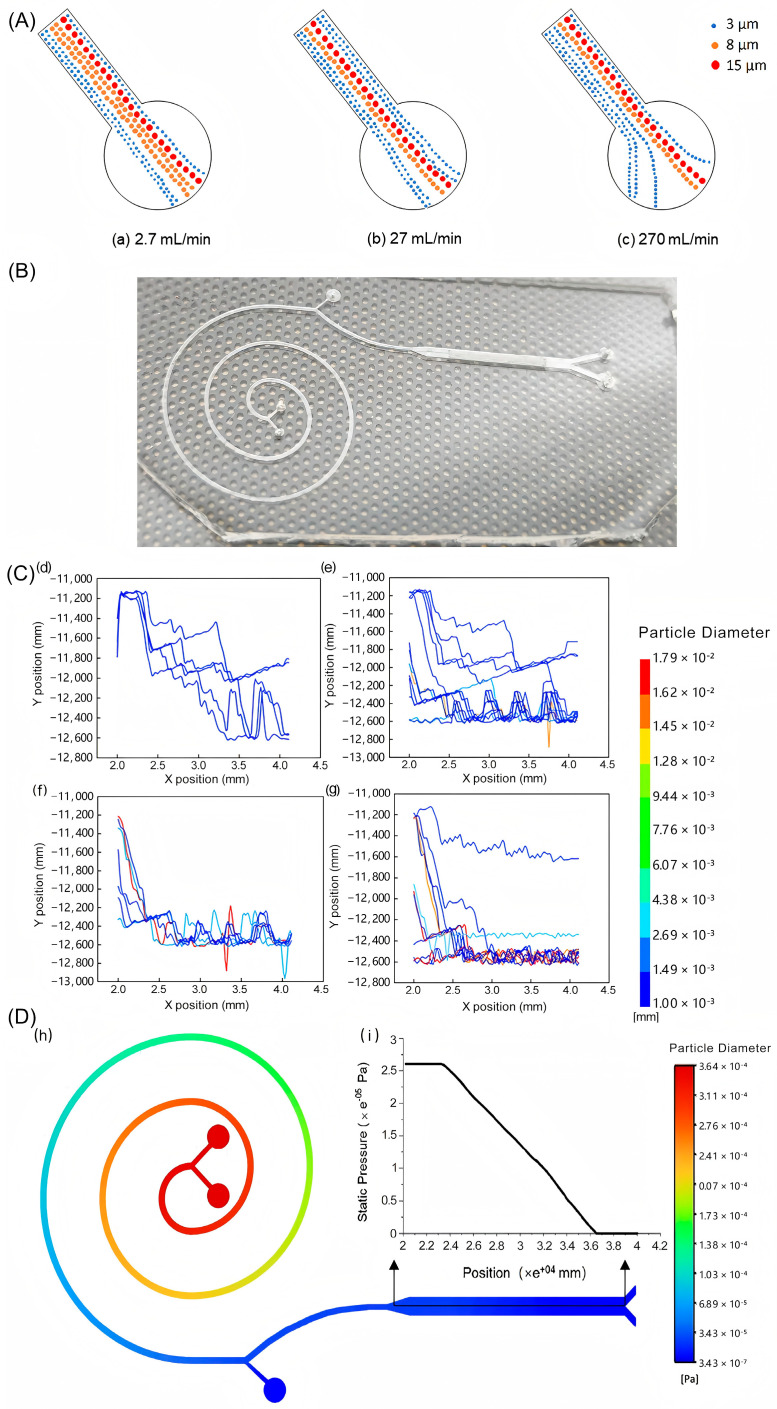
(**A**) Trajectory of blood cells in the spiral channel. (**a**–**c**) show the movement trajectories of blood cells in the spiral channel when the injection flow rates of both the blood and the buffer were 2.7 mL/min, 27 mL/min, and 270 mL/min, respectively; (**B**) photograph of a microfluidic chip for blood cell sorting; (**C**) trajectory of blood cells in the DLD channel. (**d**–**g**) show the movement trajectories of blood cells in the DLD channel when the injection speeds of both the blood and the buffer were 0.01 mm/s, 0.1 mm/s, 1 mm/s, and 10 mm/s, respectively; (**D**) the pressure contour in the microfluidic sorting chip at the flow rate of 0.1 mm/s. (**h**) Pressure distribution in the whole microchannel. (**i**) Pressure variation in the DLD channel. Data are shown for movement distance X along the bottom of the integrated chip microchannel in the direction of the longitudinal symmetry axis of the DLD.

As shown in Figure 2A, at a flow rate of 2.7 mL/min, the exit beam is relatively flat, forming a narrow stream at the spiral exit. As the injection flow rate increases, the collision between cells intensifies, and the flow stream becomes increasingly defocused. The flow beam at the outlet of the spiral channel gradually extends, and almost all cells are focused toward the equilibrium position near the inner wall, resulting in a significant increase in the focused stream width and accurate filling of the spiral outlet at a flow rate of 270 mL/min. In the spiral channel, most of the leukocytes (between 10 and 20 μm in diameter) and red blood cells (5–7 μm in diameter) are dominated by inertial force and flow mainly from spiral outlet C; most of the smaller cells (red blood cells and platelets between 2 and 4 μm in diameter) were predominantly determinant in the Dean trajectory, and large cells that were not excluded were screened for definitive lateral displacement in the second order.

The cells were trackable, as shown in Figure 2C. As the injection speed of the inlet sample decreased, the number of large cells entering the DLD channel increased. The larger blood cells (erythrocytes with diameters ranging from 5 to 7 μm and leukocytes with diameters ranging from 10 to 20 μm) are dominated by the inertial lift force, shifting downward with the flow line, and mainly exiting through spiral outlet C. The smaller blood cells had a greater chance to follow the zigzag path of collisions between microcolumns, and most remained in the lower half of the main DLD tract, with very few exiting from the upper DLD outlet. Finally, most of the cells flowed out from lower exit B of the DLD, and the serum flowed out from upper exit E of the DLD. The cell trajectory diagram visually confirms that this dual-phase microfluidic chip, which possesses an integrated DLD module and an inertial spiral module, can efficiently sort blood cells.

To confirm that the pressure at various locations in the microchannel remained within a normal range without high or low pressures, it is necessary to determine the pressure distribution throughout the chip channel. A pressure contour of the whole chip and the pressure drop variation of the DLD array are both shown in Figure 2D. Since the simulation outlet pressure was set to 0 Pa, the pressure at the DLD outlet was also close to 0 Pa. The main flow channel showed a trend of uniformly decreasing pressure as the sample flowed in. Moreover, the maximum pressure for the flow channel was at the chip inlet and the minimum pressure was at spiral outlet C, but both pressures were less than one atmospheric pressure. It suggests that at a moderate flow rate like 0.1 mm/s, the pressure in the microchannels generally did not exceed standard atmospheric pressure (1 atm). In this way, the possibility of the microchannel being damaged by excessive pressure in subsequent experiments is low.

### 3.2. The Effect of Parameter Changes on Sorting Performance

#### 3.2.1. Cross-Sectional Ratio of the Inertial Spiral Channel Exit

The full width of the spiral channel was 0.5 mm, and multiple design options existed for the width of the upper outlet…as well as for the width of the lower outlet A. These designs had to maintain a total width of 0.5 mm at the spiral exit position so that the largest cells could pass through both exits. Thus, to clarify the effect of the upper and lower exit ratios on total sorting efficiency, we designed parameter models with three different exit ratios: 0.4 mm for the upper exit and 0.1 mm for the lower exit (4:1 cross-sectional ratio); 0.35 mm for the upper exit and 0.15 mm for the lower exit (7:3 cross-sectional ratio); and 0.30 mm for the upper exit and 0.20 mm for the lower exit (3:2 cross-sectional ratio). Using these three ratios, we then simulated blood cell sorting of blood samples.

**Figure 3:**
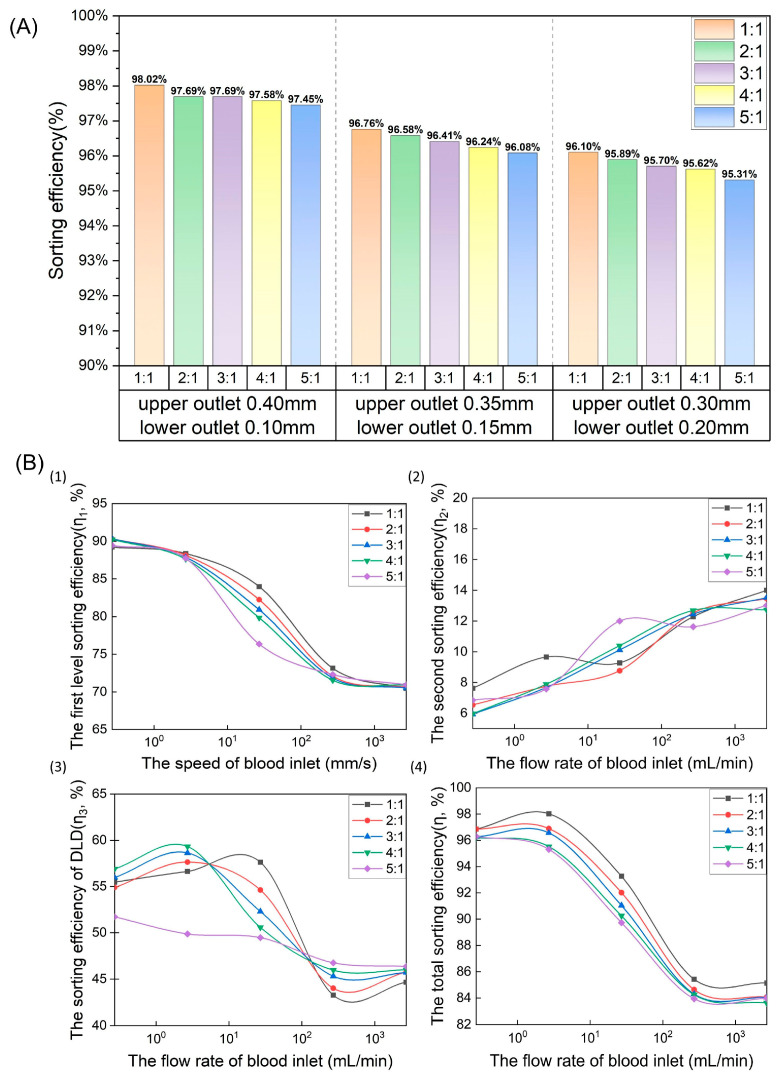
(**A**) Effect of the upper and lower cross-sectional ratio at the inertial spiral channel outlet on total sorting efficiency. Here, dark blue, orange, gray, yellow, and light blue represent different flow rate ratios for the buffer and blood at the entrance under a fixed blood of 1 mm/s; (**B**) the effect of sample injection flow rates on the sorting efficiencies. (**1**) Effect of different inlet injection flow rates and flow rate ratios on the first stage sorting efficiency; (**2**) effect of different inlet injection flow rates and flow rate ratios on the second stage sorting efficiency; (**3**) effect of different inlet injection flow rate and flow rate ratios on DLD sorting efficiency; (**4**) effect of different injection flow rates and flow rate ratios at the inlet on total sorting efficiency.

The effects of the different outlet ratios of the first stage outlet on total sorting efficiency are shown in Figure 3A. For the 0.4 mm upper and 0.1 mm lower outlet (4:1 cross-sectional ratio) model, the total sorting efficiencies of the five different inlet flow ratios were higher than those of the other two models with the same flow ratio. Thus, to improve the stability of chip performance and to realize higher total cell sorting efficiency, we adopted a spiral channel outlet design with a 0.4 mm upper outlet and a 0.1 mm lower outlet. After further data analysis, we determined that the flow rate and pressure data confirmed that this design parameter could meet the requirements of subsequent experiments.

#### 3.2.2. Flow Velocity

The main factor influencing cell aggregation and sorting behavior is the total flow rates (the sum of blood sample flow rate and buffer flow rate). Different flow rates have different effects on the deflection and sorting of blood cells: excessively low flow rate may result in problems such as poor and time-consuming cell sorting, whereas excessively high a flow rate may lead to high hydraulic pressure in the microchannel and may even break through the boundary of the microfluidic chip. Therefore, exploring the effect of various flow rates on sorting efficiency is necessary for selecting the appropriate flow rate and to verify the feasibility of the integrated solution.

The effects of the injection flow rate on sorting efficiency are shown in Figure 3B. The flow rates labeled in the figure are the blood sample injection flow rates at the inlet, including 0.27 mL/min, 2.7 mL/min, 27 mL/min, 270 mL/min, and 2700 mL/min, and the corresponding total flow rates of the mixed liquids of blood and buffer in the spiral channel are 0.1757 mm/s, 1.757 mm/s, 17.57 mm/s, 175.7 mm/s, and 1757 mm/s, respectively, when the injection flow rate ratio of the buffer to the blood sample is set as 1:1. The proportional values in the legend, such as 1:1, 2:1, 3:1, 4:1, and 5:1, represent the flow rate ratios of buffer to blood samples.

Figure 3B (1) shows that the sorting efficiency of the spiral channel gradually decreases from 90% to about 70% as the total flow rate increases from 0.27 mL/min to 2700 mL/min. This decrease in sorting efficiency with increasing flow rate is particularly evident at a blood sample flow rate of 1 mm/s: the higher the flow rate ratio of buffer to blood sample, the higher the total flow rate and the lower the sorting efficiency of the spiral channel becomes. Figure 3B (2) shows that with the increase in the total flow rate, the second sorting efficiency generally exhibits an upward trend. However, it does not represent the screening efficiency pattern of the DLD channel. Figure 3B (3) illustrates that the higher the total flow rate, the lower the sorting efficiency of the DLD channel. According to the analysis of cell trajectories in Figure 2C, when the flow rate in DLD is high, the cell trajectories have little regularity, probably due to the randomness of particle motion caused by the high-speed collision between cells and the microcolumn array in the DLD channel. With the decrease in the flow rate, the collision between the cells and microchannel gradually eased, and the trajectory of the cells in the channel showed a certain regularity. The cells moved downward gradually in the DLD channel. The total screening efficiency shown in Figure 3B (4) indicates that the screening efficiency of the chip generally shows a downward trend with the increase in the total flow rate. Only when the injection flow rate of blood at the inlet was 2.7 mL/min and flow rate ratios of the buffer to blood injection were 1:1 and 3:1 did the total sorting efficiency increase abnormally, which was the comprehensive sorting effect of the spiral channel and DLD channel. In particular, when the ratio was 1:1 and the blood sample flow rate was 2.7 mL/min, the sorting efficiency of the chip reached up to the maximum of 98.02%.

**Figure 4:**
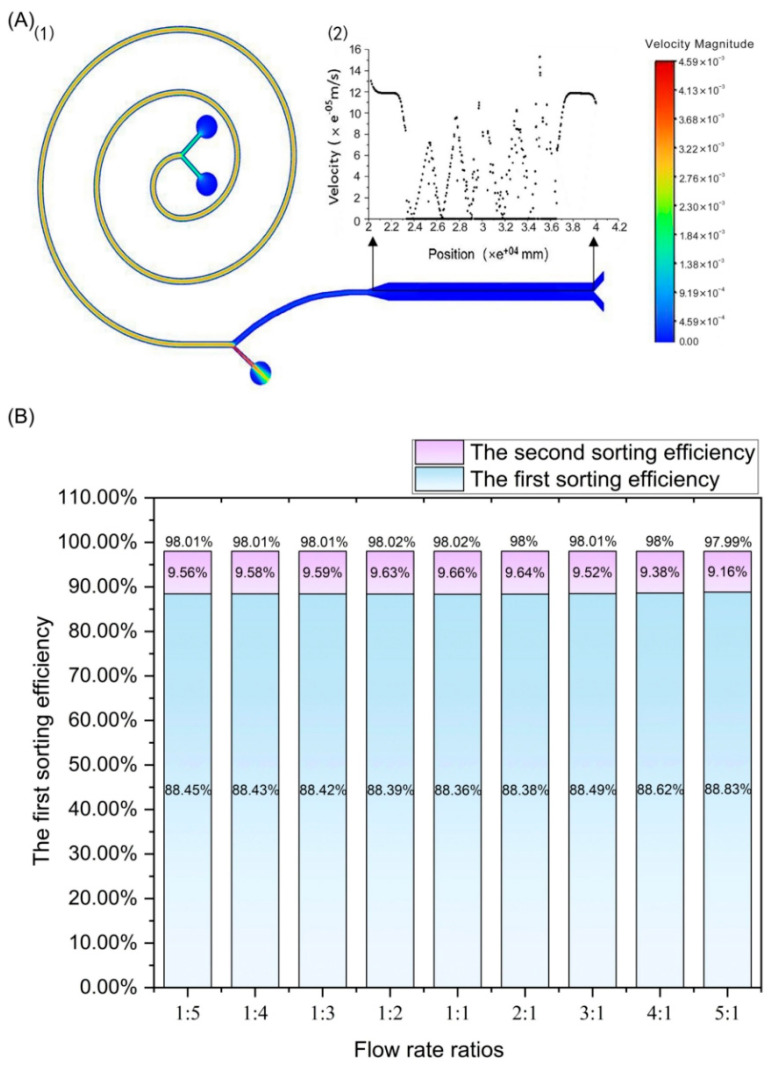
(**A**) Velocity contour in the blood cell sorting chip. (**1**) Velocity distribution in the whole microchannel; (**2**) plot showing drop variation of the cell velocity V in the DLD channel with the movement distance X. Results are shown in the direction of the longitudinal symmetry axis of the DLD. (**B**) Graph showing the effect of different flow rate ratios of the buffer to whole blood sample on sorting efficiency. Data are shown for the case in which the sum of the flow rate in inlets for the buffer and blood samples were fixed at 5.4 mL/min.

From the flow velocity contour for the whole chip shown in Figure 4A (1), it can be seen that when the injection flow rate at both inlets is 2.7 mL/min, the channel flow velocity of the spiral module is around 54 mL/min and the channel flow velocity of the DLD module is around 13.5 mL/min. As the sample flows through the main channel, overall variation in velocity remains relatively uniform, and the velocity at the outer exit of the sorting spiral can surge but will gradually return to a uniform flow rate before reaching the exit. In addition, as shown in Figure 4A (2), the velocity variation of DLD array cells along the path of the DLD array is relatively small, but obvious variation is present both before and after collisions between particles and the microcolumns.

#### 3.2.3. The Injection Rate Ratio of the Buffer to the Whole Blood Sample

When the total flow rate is fixed, the effect of changes in the buffer and whole blood flow rates ratio is mainly reflected in the concentration of blood in the main channel. Usually, when the ratio of these two flow rates decreases, the throughput of the chip increases slightly, but this will cause a decrease in sorting accuracy. Conversely, when the ratio of the two flow rates increases, the sorting accuracy is slightly improved but the throughput of the chip decreases. In addition, high blood concentrations in the channel can easily lead to device clogging, whereas low blood concentration will lead to a decrease in the concentration of proteins, nucleic acids, and other indicators in the serum collected, thereby reducing the accuracy of blood tests. Therefore, it is essential to investigate the effects of the buffer to blood sample flux ratio of this chip to determine total sorting efficiency.

The interface ratio of the upper and lower outlets of the end of the inertial spiral channel was set to 4:1, and the total velocities of the blood and buffer samples were fixed at 5.4 mL/min. Nine different simulations with different injection rate ratios of the buffer to the whole blood sample were performed. The results shown in Figure 4B indicate that changes in the flow rate ratio of the buffer to whole blood samples had little impact within 0.03% on the total sorting efficiency. The maximum total sorting efficiency reached up to 98.02% when the flow rate ratios of blood sample to buffer were 2:1, 1:1, and 1:2. Combined with Figure 3, it also shows that, for the developed blood cell sorting chip, the main influence on blood cell sorting efficiency is the flow rate rather than the flux ratio of the buffer to the blood sample. This phenomenon has also been confirmed by simulation results at several other flow rates. Table S1 in the Supplementary File shows the calculation errors in this simulation and summarizes the different injection flow rates and injection flow ratios of buffer and blood samples.

### 3.3. Experimental Results

**Polystyrene cell-like microspheres**

**Figure 5:**
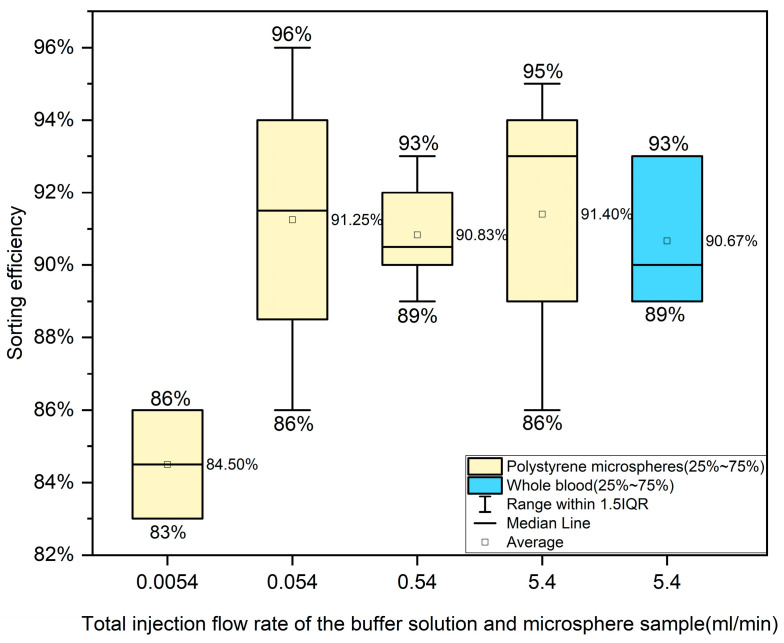
The screening efficiencies of polystyrene cell-like microspheres and whole human blood.

To evaluate the chip’s screening efficiency, red-fluo polystyrene microspheres (Shanghai Yiyuan Bio, Shanghai, China) with a diameter of 5 μm and a concentration of 10 mg/mL (1.529 × 10^10^ counts/mL) were used to simulate the motivation of red blood cells (5–7 μm) and platelets (2–4 μm), while microspheres with a diameter of 15 μm and a concentration of 10 mg/mL (5.66 × 10^8^ counts/mL) were used to simulate the trajectory of white blood cells (10–18 μm). The two types of microsphere samples were mixed with the volume ratio of 1:1, injecting a fluid into one entry of the chip while injecting the buffer at the same flow rate into another entry. The injection flow rate of one inlet ranges from 0.0027 mL/min to 2.7 mL/min (total injection flow corresponding from 0.0054 mL/min to 5.4 mL/min), which is partly within the ideal injection flow rate range found in simulation. The experimental results are shown in Figure 5. Detailed data can be found in Supplementary Table S2.

Consistent with the simulation results, the injection flow rate significantly affects the screening performance, and the optimal flow rate of 0.054 mL/min achieves a screening rate of 96%. However, the stable high screening efficiencies occur within the flow rate range of 0.054 mL/min–5.4 mL/min. Regardless of the average value (91.45%) or median value (93%), the screening efficiency of cell-like microspheres at a total flow rate of 5.4 mL/min is the highest. This trend is consistent with the simulation results, in which the highest screening efficiency also occurs at the total flow rate of 5.4 mL/min, provided that screening experiments with flow rates higher than 5.4 mL/min (such as 54 mL/min) cannot be conducted due to leakage issues. In addition, at any flow rate, the experimental screening efficiency was about 5–6% lower than the simulated screening efficiency. This performance degradation phenomenon may be due to structural defects in the device during the preparation process, fluctuations in flow rate during injections, and issues with device sealing.

**Whole human blood**

**Figure 6:**
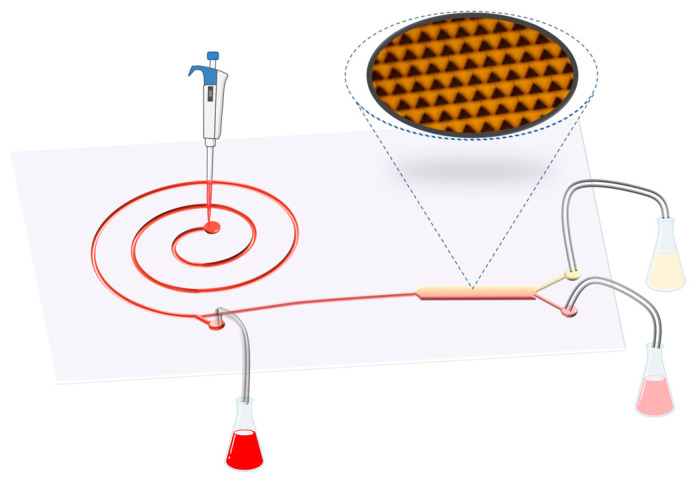
The portable operation diagram of the blood screening chip. The deep red liquid at spiral outlet C has the highest content of blood cells, followed by the red liquid at secondary outlet D. The sample at serum outlet E is a clear and transparent light-yellow color with almost no blood cells, close to serum.

For portable use, we combined two entrances A and B in the chip structure as a single entrance with a diameter of about 1 mm and conducted blood cell screening tests in the blood. The blood and buffer was prepared in a 1:1 ratio, and the blood–buffer was injected with a velocity of 5.4 mL/min, which is based on the experimental results from microsphere screening. The experimental operation is shown in Figure 6. It was found that the sample of serum at outlet E was clear and transparent light yellow, the liquid color of secondary outlet D was red, and the color at spiral outlet C was the darkest. The sorting efficiency of whole blood is displayed in the rightmost column of Figure 5. It shows an average sorting efficiency of 90.67%, slightly below that of the polystyrene microspheres, and a higher stability with a standard deviation of 2.08. Detailed experimental data can be found in Supplementary Table S3.

## 4. Discussion

With microfluidic chips, blood cells are often removed from the blood sample by designing different microchannel units according to their differences in physical properties (such as deformability, size, hydrodynamic properties, etc.). The sorting efficiency and fluxes covered in this paper range from 84% to 98%, and from 0.0054 mL/min to 5400 mL/min. When the flow rate of the blood–buffer was 0.2 mm/s, the optimal sorting efficiency could reach up to 98% and the total flux was 5.4 mL/min at this point in simulation. However, at any flow rate, the experimental screening efficiency was about 5–6% lower than the simulated screening efficiency. This sorting performance being lower than expected may be due to structural defects in the device during the preparation process, fluctuations in flow rate during injections, and issues with device sealing.

Table 1 summarizes sorting characteristics of this study and several typical model design architectures reported before for cell sorting. The sorting efficiency of the developed chip in this research can maintain the higher level compared to other reported research, while increasing flux. Furthermore, the chip can be prepared by only a single-step demolding and subsequent bonding, though it is an integrated microfluidic chip. Not only is the speed of manufacturing the chip faster, but the success rate is also higher, which can save both material and time costs. Compared to other reported studies, our research clearly balances high-throughput, screening efficiency, and manufacturing costs better.

Table 1 also shows that the screening microchannels (the last two studies) integrating both spiral and DLD have better overall screening efficiency compared to other single channels or integrated channels composed of the same types. Combined with the efficiencies in Table S1, we think this integrated chip can simultaneously utilize the advantages of spiral channels to quickly separate particle groups with large differences in size, and DLD that can use arrays and its length to more finely separate particles based on size. This research has certain enlightenment and reference significance for future studies in cell sorting and analysis as well as serum testing.

## 5. Conclusions

We developed a label-free and highly efficient screening microfluidic chip by integrating inertial and deterministic lateral displacement sorting methods to achieve blood cell separation from whole blood specimens. Both simulation and experimental results showed that the chip can achieve high efficiency in cell sorting while ensuring high throughput and low cost, which provides a convenient platform for research related to cell sorting. In addition, we also studied the effects of different microchannel structures and test conditions on cell sorting efficiency. Consequently, the following conclusions were obtained.

1. The universal chip can efficiently separate most cells with different sizes from blood samples, which enables real-time and efficient on-chip serum testing to be achieved.

2. The cross-sectional ratio of the upper outlet to the lower outlet in inertial spiral channel affects the cell sorting. In a certain range, increasing this ratio can effectively improve the sorting efficiency. Moreover, the total flow rate in the microchannel, rather than the flux ratio of the buffer to the blood sample, played a vital role in influencing the sorting efficiency of the microfluidic chip.

3. For this device, when the injection flow rate of the blood sample is fixed at 5.4 mL/min, an injection speed ratio of 1:1 for the blood sample and the buffer achieves the highest cell separation efficiency, which showed ~96% in simulation and ~91% in experiments.

## Figures and Tables

**Table 1:** Summary of microfluidic cell sorting methods and their performance.

Microchannel	Fluid Characteristics	Flow Rate	Sample	Sorting Efficiency	References
double spiral	inertia	170–1000	blood; polystyrene particles	90–95%	Sun, J. et al. [23].
DLD	inertia	≥10 mL/min	blood	85%	Loutherback, K. et al. [24].
double spiral	inertia	2.3 mL/min	blood	≥92%	Jeon, H. et al. [25].
capture pools	magnetic bead	8 mL/min	blood	84.5%	Lemaire, C. A. et al. [26].
DLD	inertia	1.6–16 mL/min	cancer cells (not blood)	90%	Liu, Z. et al. [27].
single spiral	inertia	0.1–1 mL/min	polystyrene particles	—	Al-Halhouli, A. et al. [28].
double spiral	inertia	170–250 μL/min	blood	92%	Sun, J. et al. [20].
integration	inertia	400 μL/min	tumor cells (not blood)	99.9%	Xiang, Nan. et al. [13].
**i** **ntegration**	**inertia**	**5.4 mL/min**	**simulation**	**96–98%**	**This study**
			**polystyrene micropheres**	**90~96%**	
			**blood**	**90~93%**	

## Data Availability

The data that support the findings of this study are available from the corresponding authors upon reasonable request.

## Supplementary Materials

The following supporting information can be downloaded at: https://www.mdpi.com/article/10.3390/bioengineering12121326/s1, See the supplementary material for more details about experimental methods and effects of some variables on sorting. Table S1: Three sorting efficiencies and their standard deviations; Table S2: Experimental results of the sorting efficiencies of cell-like microsphere samples; Table S3: Data from sorting experiment of the whole blood samples.
