# Supplementary material for: A Universal and Single-Step (De)Molding Sorting Chip Integrating Inertial and Deterministic Lateral Displacement Units
Source: Bioengineering (Basel). 2025 Dec 5;12(12):1326. doi: 10.3390/bioengineering12121326 (PMC12730075; doi:10.3390/bioengineering12121326)
Supplement: Supplementary file 1 [file bioengineering-12-01326-s001.zip › bioengineering-3961504-supplementary.pdf]

Supplementary Material for

A universal and single-step (de)molding sorting chip integrating inertial and deterministic lateral displacement units

Yifan Guo[1, †], Xiaoyu Qu[1, †], Zhaogang Dong[2], Mengmeng Xiao[3, *], and Jingjing Xu[1, *]

[1] Shandong Key Laboratory of Next-Generation Semiconductor Technology and Systems, School of Integrated Circuits, Shandong University, Jinan 250100, China

[2] Department of Clinical Laboratory, Qilu Hospital of Shandong University, Jinan, 250100, P.R. China

[3] Carbon-based electronics research center, Peking University, Beijing 100871, P.R. China

† The authors contributed equally.

* Corresponding: xujj@sdu.edu.cn, mmxiao@pku.edu.cn

This file includes:

Supporting text

Tables S1 to S2

Support text

Table S1: Three sorting efficiencies and their standard deviations

Table S1 shows the calculation errors in this simulation work and lists the three sorting efficiencies and their standard deviations under different injection flow rates and different injection flow ratio of buffer and blood samples. The average and standard errors here are obtained on the basis of the simulation results of three independent experiments, which is helpful for us to analyze the regularity and reliability of the current simulation data.

Table S1: Three sorting efficiencies and their standard deviations.

Flow rate	Flow rate ratio of buffer to blood	Total sorting efficiency η	The standard deviation of η	The sorting efficiency of spiral module η_1	The standard deviation of η_1
0.27 mL/min	1:1	96.03%	1.53	89.22%	0.00
0.27 mL/min	2:1	96.61%	0.21	90.33%	0.02
0.27 mL/min	3:1	95.39%	1.68	89.62%	0.61
0.27 mL/min	4:1	96.30%	0.08	89.62%	0.54
0.27 mL/min	5:1	96.29%	0.00	89.44%	0.00
2.7 mL/min	1:1	97.07%	0.96	88.36%	0.00
2.7 mL/min	2:1	95.89%	0.00	88.14%	0.00
2.7 mL/min	3:1	95.69%	0.00	87.87%	0.01
2.7 mL/min	4:1	95.52%	0.00	87.63%	0.00
2.7 mL/min	5:1	95.34%	0.02	87.49%	0.21
27 mL/min	1:1	93.26%	0.00	83.98%	0.00
27 mL/min	2:1	92.02%	0.00	82.25%	0.00
27 mL/min	3:1	91.04%	0.00	80.92%	0.00
27 mL/min	4:1	90.25%	0.00	79.85%	0.00
27 mL/min	5:1	89.65%	0.07	77.77%	1.32
270 mL/min	1:1	85.43%	0.01	73.14%	0.01
270 mL/min	2:1	84.69%	0.04	71.94%	0.15
270 mL/min	3:1	84.34%	0.03	71.73%	0.12
270 mL/min	4:1	84.21%	0.03	71.68%	0.10
270 mL/min	5:1	84.18%	0.11	71.95%	0.31
2700 mL/min	1:1	84.90%	1.52	70.48%	0.02
2700 mL/min	2:1	83.94%	0.11	70.51%	0.09
2700 mL/min	3:1	84.02%	0.35	70.44%	0.07
2700 mL/min	4:1	83.65%	0.03	70.61%	0.24
2700 mL/min	5:1	82.47%	2.21	70.65%	0.26

Table S2: Experimental results of the sorting efficiencies of cell-like microsphere samples.

Table S2 shows the screening efficiency of different injection volume ratios for various flow rates, buffers, and microsphere samples. The results are consistent with the simulations. The injection flow rate significantly affects the screening performance, and the optimal flow rate of 0.027 mL/min achieves a screening rate of 96%. However, the stable high screening efficiencies occurs within the flow rate range of 0.027 ml/min - 2.7 ml/min.

Table S2: Experimental results of the sorting efficiencies of cell-like microsphere samples.

Number	Buffer injection velocity of flow	Injection flow rate of cell-like microsphere sample	Sorting efficiency	Average value & standard deviation
1	0.0027mL/min	0.0027mL/min	Backflow occurs	
2	0.0027mL/min	0.0027mL/min	Backflow occurs	84.5%
3	0.0027mL/min	0.0027mL/min	86%	2.12
4	0.0027mL/min	0.0027mL/min	83%	
5	0.027mL/min	0.027mL/min	91%	
6	0.027mL/min	0.027mL/min	96%	91.25%
7	0.027mL/min	0.027mL/min	92%	4.11
8	0.027mL/min	0.027mL/min	86%	
9	0.27mL/min	0.27mL/min	90%	
10	0.27mL/min	0.27mL/min	91%	
11	0.27mL/min	0.27mL/min	Leakage occurs	
12	0.27mL/min	0.27mL/min	90%	90.83%
13	0.27mL/min	0.27mL/min	93%	1.47
14	0.27mL/min	0.27mL/min	92%	
15	0.27mL/min	0.27mL/min	Leakage occurs	
16	0.27mL/min	0.27mL/min	89%	
17	2.7mL/min	2.7mL/min	Leakage occurs	
18	2.7mL/min	2.7mL/min	86%	
19	2.7mL/min	2.7mL/min	Leakage occurs	
20	2.7mL/min	2.7mL/min	93%	
21	2.7mL/min	2.7mL/min	89%	91.4%
22	2.7mL/min	2.7mL/min	95%	3.78
23	2.7mL/min	2.7mL/min	Leakage occurs	
24	2.7mL/min	2.7mL/min	94%	
25	2.7mL/min	2.7mL/min	Leakage occurs	
26	27mL/min	27mL/min	Leakage occurs	
27	27mL/min	27mL/min	Leakage occurs	/
28	27mL/min	2.7mL/min	Leakage occurs	

Table S3: Data from sorting experiment of the whole blood samples.

Table S3 shows the experimental data when screening whole blood samples using the chip at 2.7ml/min.

Table S3: Data from sorting experiment of the whole blood samples.

Number	Injection velocity of the blood-buffer flow	Sorting efficiency	Average value & standard deviation
1	5.4 mL/min	Leakage occurs	90.67%<br>2.08
2	5.4 mL/min	93%	
3	5.4 mL/min	90	
4	5.4 mL/min	Leakage occurs	
5	5.4 mL/min	89%	
6	5.4 mL/min	Leakage occurs	
7	5.4 mL/min	Leakage occurs	
